# The preprotein translocase YidC controls respiratory metabolism in *Mycobacterium tuberculosis*

**DOI:** 10.1038/srep24998

**Published:** 2016-05-11

**Authors:** Preeti Thakur, Nagavara Prasad Gantasala, Eira Choudhary, Nirpendra Singh, Malik Zainul Abdin, Nisheeth Agarwal

**Affiliations:** 1 Translational Health Science and Technology Institute, NCR Biotech Science Cluster, 3rd Milestone, Faridabad–Gurgaon Expressway, Faridabad 121001 India; 2 Faculty of Science, Jamia Hamdard, Hamdard Nagar, New Delhi-110062, India; 3 Regional Center for Biotechnology, NCR Biotech Science Cluster, 3rd Milestone, Faridabad–Gurgaon Expressway, Faridabad- 121001 India; 4 Symbiosis School of Biomedical Sciences, Symbiosis International University, Lavale, Pune- 412115 (Maharashtra) India

## Abstract

The YidC–Oxa1–Alb3 preprotein translocases play a vital role in membrane insertion of proteins in eukaryotes and bacteria. In a recent study we observed that *Rv3921c*, which encodes putative YidC translocase in *Mycobacterium tuberculosis* (Mtb), is essential for *in vitro* growth of bacteria. However, the exact function of this particular protein remains to identify in mycobacterial pathogens. By performing a systematic study here we show that YidC of Mtb is an envelope protein, which is required for production of ATP and maintenance of cellular redox balance. Drastic effects of depletion of Rv3921c on the expression of hypoxic genes, ATP synthases, and many proteins of central metabolic and respiratory pathways shed a significant light on the function of YidC towards controlling respiratory metabolism in Mtb. Association of YidC with proteins such as succinate dehydrogenases and ubiquinol-cytochrome C reductase further confirms its role in respiration. Finally we demonstrate that YidC is required for the intracellular survival of Mtb in human macrophages.

Membrane proteins (MPs) that constitute ~30% of total proteins in a cell^1^,^2^ are vital for energy metabolism, transport and cellular signaling in different life forms. Assembly of MPs into the lipid bilayer is accomplished by two major translocation machineries known as Sec (general secretion) and Tat (twin arginine translocation) that translocate proteins in unfolded and folded forms, respectively^3^,^4^,^5^. Translocation of MPs in mitochondria, which lack Sec or Tat translocation machinery in most of the organisms except in protist *Reclinomonas americana* and associated protozoa^6^,^7^, remained unknown until 1994 when it was reported that a mutation in a new protein called Oxa1 (for oxidase assembly factor) affects the biogenesis of cytochrome c oxidase^8^,^9^ and formation of the F1F_o_-ATP synthase^10^. The Oxa1 belongs to YidC/Oxa1/Alb3 family of proteins which was found to be essential for translocation of Cox2 and various other MPs from mitochondrial matrix to inner membrane^11^,^12^,^13^. Oxa1 counterpart in chloroplasts, known as Alb3, is required for the post-translational insertion of the light-harvesting chlorophyll-binding proteins into thylakoids^14^.

A comparative amino acid sequence analysis of Alb3 further revealed significant similarity with a bacterial inner membrane protein, later annotated as YidC, in *Pseudomonas putida*, *Escherichia coli*, *Coxiella burnetii*, *Bacillus subtilis* and a cyanobacterium *Synechocystis* sp. strain PCC6803^15^. In *E. coli*, YidC is a 61 kDa protein of the inner membrane which comprises of 6 transmembrane (TM) regions and a large periplasmic domain at the N-terminus between TM1 and TM2^16^. Homology search indicates high sequence similarity among YidC proteins of Gram-negative bacteria, which unanimously exhibit the presence of 6 TM regions^17^. In contrast, the YidC homologues of Gram-positive bacteria distinctly contain only 5 TM segments and short periplasmic N-terminal tails with a cleavable signal sequence^18^. Analysis of *yidC* locus in *E. coli* reveals that it is transcribed in an operon with neighboring genes arranged in an order of *rpmH, rnpA, yidD, yidC* and *trmE* that directly influence the function of YidC^19^. YidC is essential for the growth of *E. coli* and its depletion results in the induction of several stress proteins such as the phage shock protein PspA, and chaperones ClpB, DnaJ, DnaK, GroEL, HtpG, HflC and HflK^20^,^21^,^22^,^23^. MPs that are solely dependent on YidC for their insertion include F_o_c subunit of F_1_F_o_ ATP synthase, the mechanosensitive channel protein MscL, the phage proteins M13 procoat and Pf3 coat, and a C-tailed anchor protein TssL^24^,^25^,^26^,^27^,^28^,^29^.

*Mycobacterium tuberculosis* (Mtb), the etiological agent of most cases of tuberculosis (TB) in humans, is responsible for 1.5 million deaths annually^30^. Mtb has a unique capacity to replicate in macrophages, and it is extremely difficult for the host cells to clear the invading pathogens^31^. Mycobacterial proteins exported from the cytoplasm to the cell surface of bacteria or into the host play an important role in host-pathogen interaction and long-term intracellular survival of bacteria^32^. Majority of protein export in Mtb is accomplished by the involvement of the Sec and the Tat export pathways^33^. Additionally, Mtb also exhibits few specialized protein export systems that include the SecA2-dependent and Esx, also referred as Type VII, secretion systems. It is noteworthy to mention that many of these proteins are essential for *in vitro* growth of Mtb as well as for its virulence and intracellular survival^34^,^35^,^36^. Despite significant progress in characterizing the mycobacterial transport machineries the underlying mechanism of protein export by Sec or Tat translocases is largely uncharacterized in mycobacteria. Moreover, there is a complete lack of information on the role of YidC insertase in mycobacteria. Comparative sequence analysis reveals that Rv3921c, which is annotated as probable conserved transmembrane protein in the Tuberculist database (http://tuberculist.epfl.ch/index.html), exhibits ~17% identity with YidC2 of *Bacillus halodurans*, a Gram-positive bacterium (Supplementary Fig. 1). Interestingly, *Rv3921c* and other neighboring genes in its locus are highly conserved among different mycobacterial species. These observations thus strongly suggest an important role of this protein in mycobacterial physiology.

In this study we show that YidC is a cell envelope-localized protein in Mtb whose expression is regulated by membrane potential. Transcriptional and proteomic analyses reveal that YidC regulates the ATP production in Mtb as evidenced by modulation of genes related to central metabolism and respiration in cells depleted with YidC. Significantly reduced levels of ATP, elevated NADH/NAD^+^ ratio and perturbed membrane potential in YidC depletion strain further corroborate its role in controlling the energy-generating metabolic pathways in Mtb. Furthermore, direct interaction of YidC with electron transport system proteins succinate dehydrogenase (Rv0247c-Rv0248c) and ubiquinol-cytochrome C reductase subunit QcrA (Rv2195) are reminiscent of its involvement in biogenesis of respiratory chain complexes in Mtb. Finally we show that YidC is required for the intracellular growth of Mtb in human monocyte-derived macrophages.

## Results

### YidC is localized to cell envelope of Mtb

**Figure 1:**
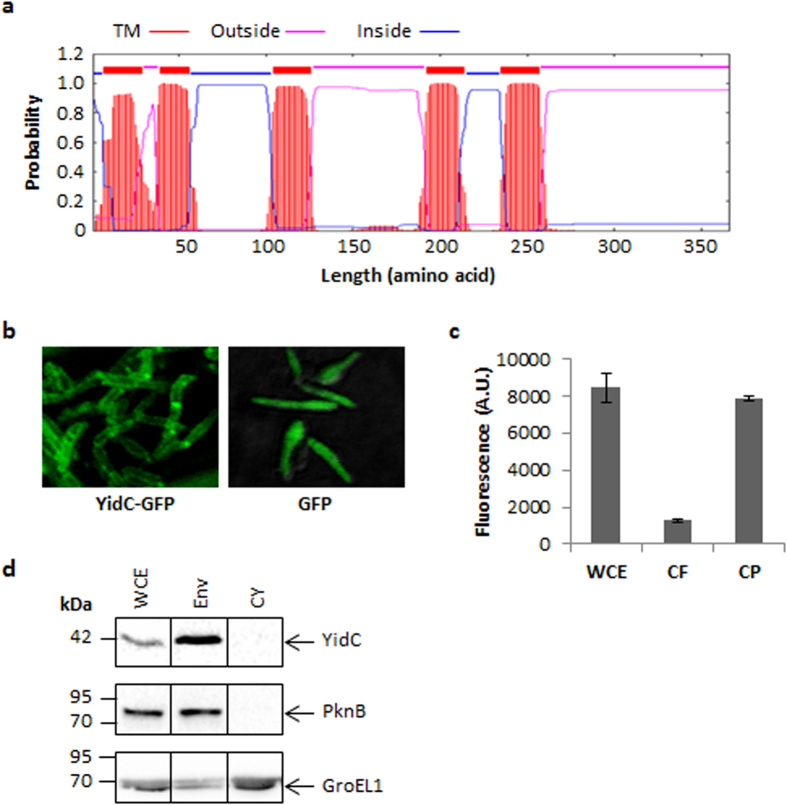
Localization of YidC in mycobacterium. (**a**) Prediction by TMHMM server v2.0^52^ reveals the presence of 5 TM regions (red) and two cytosolic loops (blue) in YidC indicating its localization in cell envelope. (**b**) Fluorescent microscope analysis of *M. smegmatis* expressing YidC-GFP fusion protein or GFP alone shows that YidC-GFP and not the GFP is confined to cell envelope. (**c**) Absence of fluorescence in culture filtrate (CF) despite substantial fluorescence in whole cell extract (WCE) and cell pellets (CP) of *M. smegmatis* expressing YidC-GFP fusion protein indicates that YidC is not secreted outside the cell. (**d**) Cell envelope localization of YidC was also confirmed in wild-type Mtb by subjecting equal amount (20 μg) of proteins from the different subcellular fractions to anti-YidC immunoblotting. Antibodies against PknB which is localized in cell envelope (Env) or GroEL1 which is present in both the cell envelope and cytoplasm (CY) were simultaneously used to assess the purity of fractions. Data represent two experiments in (**b**,**d**). Proteins from different subcellular fractions were resolved on denatured polyacrylamide gel and blotted under the same experimental conditions in (**d**). Mean + s.d. of three measurements is shown in (**c**).

With the help of six TM segments, the YidC in *E. coli* is localized to inner membrane. Contrary to *E. coli* protein, YidC of Mtb exhibits only 5 TM segments, lacking the sixth TM region which is present towards the C-terminus in *E. coli* counterpart. Moreover, in Mtb YidC lacks a large periplasmic domain which is present between TM1 and TM2 of *E. coli* protein (Fig. 1a). As a result, YidC of Mtb is much smaller in size (366 aa) in comparison to its *E. coli* counterpart (548 aa).

To gain an insight into the function of YidC in Mtb, we sought to determine its localization in mycobacterium. A 1098 bp DNA sequence corresponding to *yidC* open reading frame (ORF) was cloned upstream to a gene encoding green fluorescent protein (GFP) in *E. coli*-mycobacteria shuttle plasmid, pTetR^37^ (Supplementary Table 1) for the conditional expression of YidC as C-terminal GFP fusion. Simultaneously, cells expressing GFP alone were used as control. Localization of GFP was subsequently analyzed in bacteria treated with inducer anhydrotetracycline (ATc), under the fluorescent microscope. Our results indicated that while GFP alone is uniformly distributed, the fluorescence obtained from YidC-GFP is restricted to periphery of bacteria suggesting localization of YidC in cell envelope (Fig. 1b). Moreover, absence of fluorescence in culture filtrate of YidC-GFP expressing bacteria rules out secretion of YidC in the extracellular environment (Fig. 1c). Next, to analyze localization of native YidC protein in Mtb, different subcellular fractions of wild-type Mtb were prepared and subjected to immunoblotting by using anti-YidC antibodies. It was observed that YidC is specifically present in the cell envelope and not in the cytoplasm of Mtb (Fig. 1d and Supplementary Fig. 2).

### Effect of YidC depletion on whole-genome transcriptional profile of Mtb

**Figure 2:**
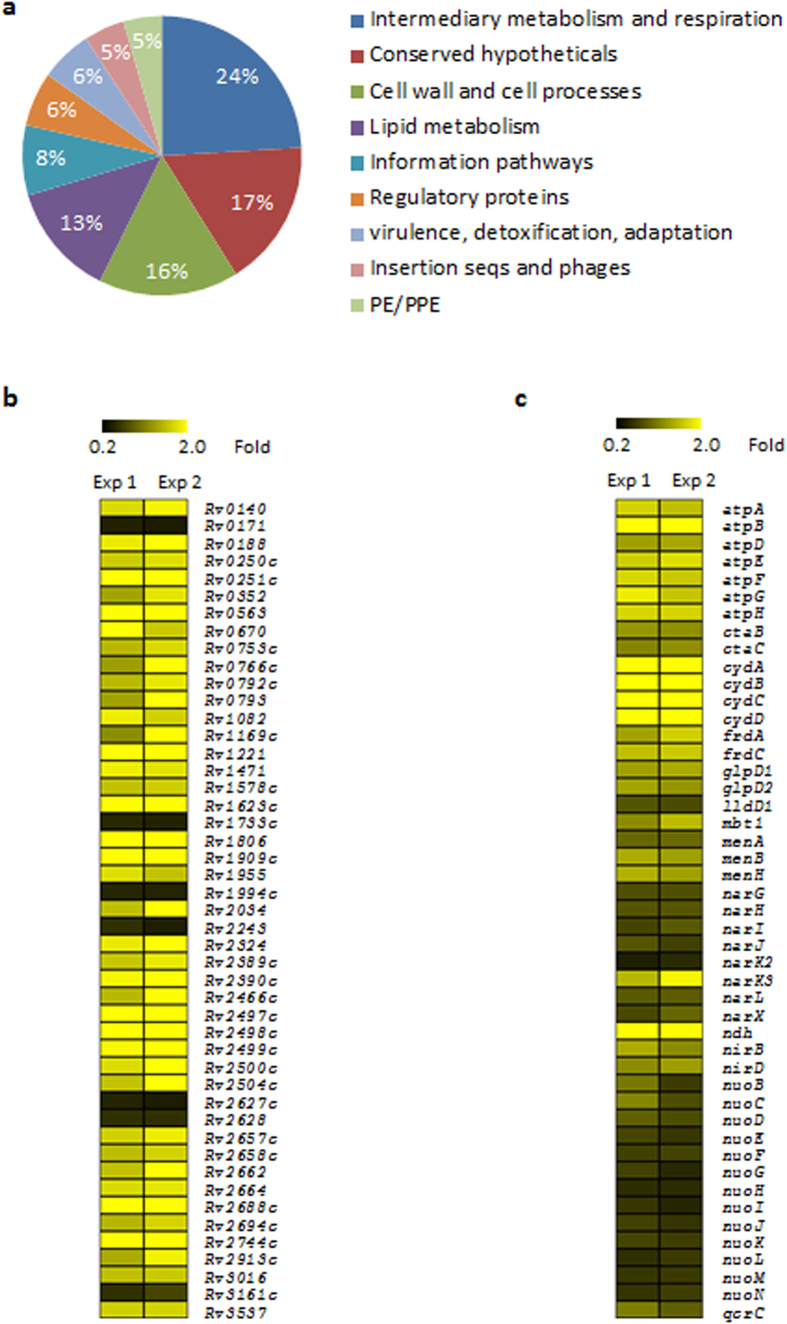
Transcriptional response of Mtb to YidC depletion. (**a**) Effect of YidC depletion on global changes in gene expression of Mtb and contribution of genes from different metabolic categories. A total of 381 genes modulated by >1.6-fold (*P* < 0.05, as determined by Student’s t-test) were grouped according to the classification by Tuberculist database. (**b**,**c**) Heat map displaying the expression levels of genes belonging to EHR (**b**) and respiration (**c**) in Mtb depleted with YidC. The color scales represent fold change in expression of a gene in YidC-depleted strain relative to control Mtb. Data represent two independent microarray experiments (Exp 1 and Exp 2), each containing triplicate values for 3924 genes in (**b**,**c**). A complete list of differentially expressed genes is provided in Supplementary Data 1.

Localization of YidC to cell surface suggests that it may have a signalling role in mycobacteria. Growth defect in Mtb lacking YidC^37^ further proposes that depletion of YidC results in altered expression of core metabolic pathways. In order to examine the cellular response to YidC depletion in Mtb, global transcriptional profiles of dCas9-expressing cells containing empty plasmid pGrna (control) or pGrna-*yidC* (*yidC*(*−*))^37^ (Supplementary Table 1) were analyzed by microarray after 4 days of YidC depletion as described in Methods. It was observed that YidC depletion modulates the expression of a total of 381 genes by >1.6-fold. Notably, expression levels of 53 upregulated and 29 downregulated genes are altered by >2.0-fold in *yidC*(*−*) (Supplementary Data 1). Interestingly, majority of the altered genes are involved in intermediary metabolism and respiration (24%), cell wall and cell processes (16%) and lipid metabolism (13%) (Fig. 2a). In addition, suppression of YidC also alters the expression of several transcriptional and translational genes (Supplementary Data 1). Further clustering of differentially expressed genes by their function reveals that ~20% of genes controlling the enduring hypoxic response (EHR) of Mtb^38^,^39^ exhibit altered expression in *yidC*(*−*) strain of Mtb (Fig. 2b). Notably, YidC depletion also causes a mild change in the expression levels of a significant number of genes (n = 73) that regulate the energy metabolism and respiration in mycobacteria (Supplementary Data 1 and Fig. 2c). Genes encoding ATP synthases (*atpA–H*), cytochrome D ubiquinol oxidases (*cydA–D*), fumarate reductases (*frdA–C*), glycerol-3-phosphate dehydrogenases (*glpD1* and *glpD2*), type II NADH dehydrogenase (NDH-2; *ndh*) and those involved in menaquinone biosynthesis (*menB* and *menH*) are upregulated, whereas genes encoding nitrate reductases (*narG-J, narL* and *narX*) and type I NADH dehydrogenases (NDH-1; *nuoB-N*) are downregulated in Mtb depleted with YidC (Fig. 2c and Supplementary Data 1). Importantly, validity of the microarray results was also confirmed by real-time quantitative reverse transcription PCR (qRT-PCR) using primers specific to some of the differentially expressed genes (Supplementary Fig. 3).

### Effect of YidC depletion on Mtb proteome

**Figure 3:**
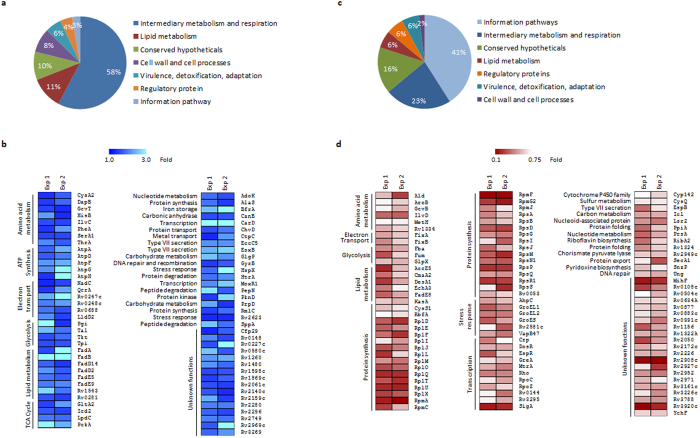
Effect of YidC depletion on proteome profile of Mtb. (**a**,**c**) Functional categories of proteins differentially expressed in *yidC*(*−*). YidC depletion results in upregulation of 71 (**a**,**b**) and downregulation of 100 proteins (**c**,**d)** by 1.3-fold or more. (**b**,**d**) Heat map represents upregulated (**b**) and downregulated (**d**) proteins that are grouped on the basis of assigned functions. Color intensities in the heat map represent levels of expression. Expression values from two independent experiments (Exp 1 and Exp 2) are shown in (**b**,**d**). All detected and identified proteins are listed in Supplementary Data 2.

Analysis of the above microarray results specifies a plausible role of YidC in regulation of respiration in mycobacteria. Next, we investigated the effect of YidC depletion on global expression of proteins in Mtb. Quantitative proteomics by isobaric tag for relative and absolute quantitation (iTRAQ) was performed with the whole cell extracts (WCEs) of *yidC*(*−*) and control strains of Mtb H_37_Rv after 4 days of depletion as described in Methods. The iTRAQ data from two individual experiments identified 1259 and 679 proteins, respectively. It was observed that YidC depletion results in upregulation of 71 proteins and downregulation of 100 proteins by >1.3-fold (Fig. 3 and Supplementary Data 2). Importantly, ~45% of upregulated (n = 31) and 50% of downregulated (n = 49) proteins exhibit >2-fold change in their levels due to YidC depletion (Supplementary Data 2). Moreover, 58% of the upregulated proteins belong to intermediary metabolism and respiration category (Fig. 3a), whereas a significant number of proteins that are suppressed in the *yidC*(*−*) control transcription and protein synthesis (Fig. 3c). Importantly, suppression of YidC in Mtb leads to accumulation of ATP synthases (AtpA, AtpD, AtpF, AtpG and AtpH) and electron transport proteins (QcrA, Rv0247c, Rv0248c, Rv0688 and LldD2) (Fig. 3b). In contrast, a number of ribosomal proteins (RplD, RplF, RplI, RplJ, RplL, RplM, RplO, RplQ, RplT, RplU, RplX, RpmA, RpmC, RpmF, RpmG2, RpsA, RpsB, RpsD, RpsG, RpsI, RpsJ, RpsM, RpsN1, RpsP, RpsQ, RpsR1, RpsS), RNA polymerase subunits (RpoC and RpoZ), transcription regulators (SigA, Rho, Crp, DosR, EspR, GreA, MtrA, Rv0144 and Rv3295) and stress response regulators (AhpC, GroEL1, GroEL2, GroES, Rv2581c and VapB47) are downregulated in *yidC*(*−*) relative to control (Fig. 3d). Several other proteins including those involved in DNA replication, transport of proteins and metals, iron storage, degradation of proteins and peptides, and metabolism of nucleotides, amino acids, carbon and lipid also exhibit differential expression in *yidC*(*−*) compared to the control Mtb (Fig. 3). Importantly, specific effect of YidC depletion on expression of these proteins was also validated by immunoblotting using specific antibodies. When we expressed Rv0248c encoding a putative succinate dehydrogenase protein fused to FLAG tag at C-terminus in wild-type and *yidC*(*−*) strains, ~4.2-fold increase in Rv0248c protein levels was observed in the WCE of *yidC*(*−*) compared to that in the wild-type bacteria (Supplementary Fig. 4a). In contrast, the immunoblotting of lysates with anti-GroEL1 antibodies revealed ~3-fold reduction in expression of GroEL1 in the *yidC*(*−*), whereas a comparable expression of an unrelated protein PknB in both the control and the *yidC*(*−*) strains indicates the equal loading of samples (Supplementary Fig. 4c).

### YidC is required to maintain ATP levels and intracellular redox balance in Mtb

**Figure 4:**
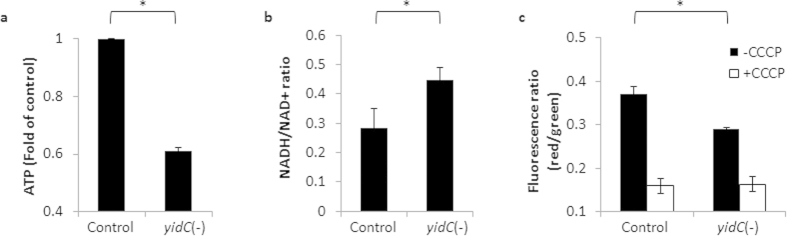
Effect of YidC depletion on cellular redox balance. (**a**–**c**) Effect of YidC depletion on cellular ATP levels (**a**), NADH/NAD^+^ ratio (**b**) and membrane potential (**c**), relative to control Mtb. Both the control and *yidC*(*−*) strains of Mtb cultured for 4 days in 7H9-OADS medium containing 50 ng/ml ATc were used. ATP was measured as described in Methods. For estimating NADH/NAD^+^ ratios, cell extracts were prepared by heat lysing cells at 55 °C for 10 minutes either in 0.2 M HCl (For NAD^+^ extraction) or 0.2 M NaOH (For NADH extraction). NADH and NAD^+^ levels were measured using yeast alcohol dehydrogenase typically as described earlier^53^. Membrane potential was determined using BacLight Bacterial Membrane Potential Kit (Molecular Probes) with 1 ml bacterial cultures at OD_600_ of 1.0 in the absence or the presence of ionophore CCCP. Briefly, cells were incubated with 30 μM of carbocyanine dye 3,3′-diethyloxacarbocyanine iodide (DiOC_2_) with or without 5 μM CCCP. Stained cells were analyzed using 488-nm excitation and emission at 528-nm (green) and 620-nm (red), respectively. The DiOC_2_ red:green ratios were calculated using mean fluorescence intensities that provide a measure of membrane potential. Mean ± s.d. of three experiments is shown in (**a**–**c**). Asterisks describe levels of significance (*P* < 0.05).

Recently it was reported that Mtb treated with ATP synthase inhibitor bedaquilline exhibits similar transcript and protein profiles as we observed with the Mtb *yidC*(*−*) strain^40^. Both bedaquilline treatment and depletion of YidC modulate the expression of hypoxic genes, ATP synthases, cytochrome D ubiquinol oxidases and ribosomal proteins to comparable levels, which strongly propose that YidC is likely required by Mtb for maintaining the cellular ATP pool. Estimation of intracellular ATP concentrations indeed supports this hypothesis and shows that in the absence of *yidC*, ATP levels are reduced by 40% in Mtb (Fig. 4a). Importantly, this effect is specific to YidC as suppression of downstream gene to *yidC*, *Rv3920c* does not impact the ATP levels in Mtb (Supplementary Fig. 5). Further, not only the ATP levels, but the intracellular NADH/NAD^+^ ratios and membrane potential are also perturbed in the *yidC*(*−*) compared to their levels in the control cells (Fig. 4b,c). Figure 4 shows that depletion of YidC shifts the NADH/NAD^+^ redox balance towards a reducing state (Fig. 4b) which also moderates the membrane potential (Fig. 4c). Overall, these results establish for the first time, a distinct role of YidC in controlling respiration in Mtb.

### Effect of YidC depletion on the expression of envelope proteins

**Figure 5:**
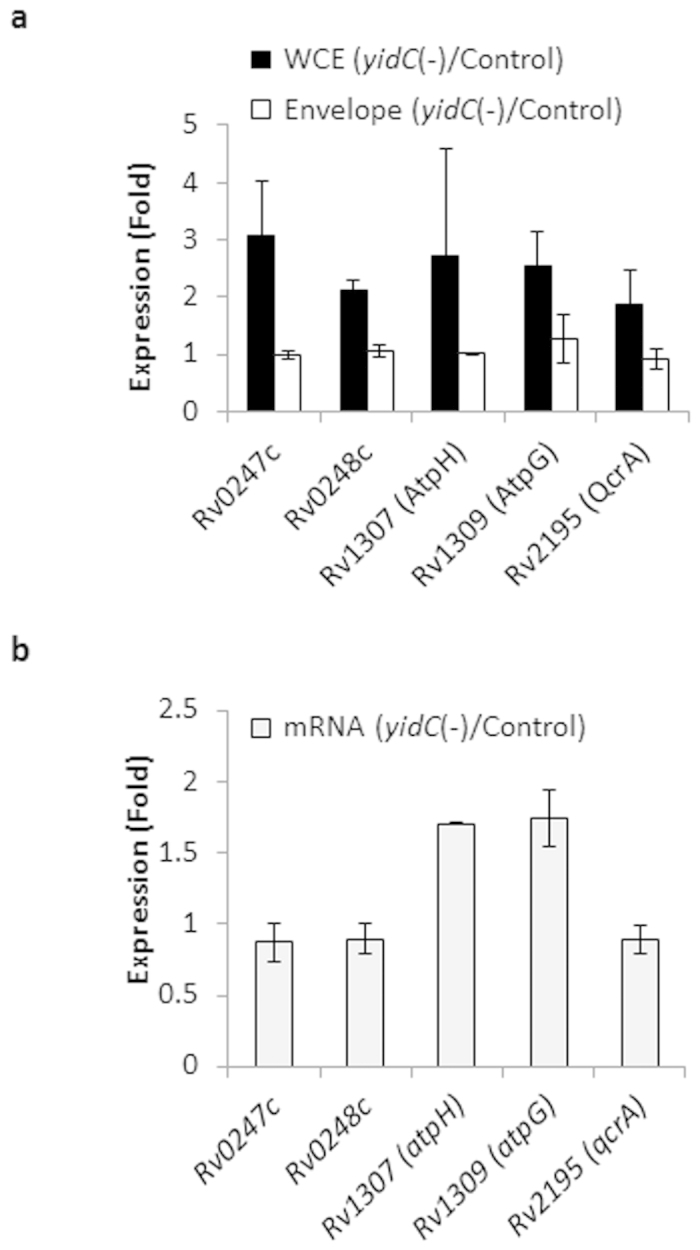
Effect of YidC depletion on the expression of envelope proteins in Mtb. (**a**) Shown are proteins that are differentially expressed in *yidC*(*−*) compared to the control Mtb strain. Those involved in respiration are depicted in bar graph, whereas a complete list is provided in Supplementary Data 3. Mean ± s.d. of two experiments is shown. (**b**) Bar graph shows microarray expression levels of selected transcripts whose protein expressions are mentioned in (**a**). Mean ± s.d. of two microarray experiments is shown. For details of differentially expressed genes please refer to Supplementary Data 1.

Although the direct effect of YidC depletion on cellular ATP levels has not been investigated earlier, its involvement in the translocation of F_o_c subunit of F1F_o_ ATP synthase in *E. coli*^29^,^41^ suggests a universal role of YidC in regulating the respiratory pathway. To understand if depletion of YidC affects the translocation of respiratory proteins in Mtb, we compared the control and *yidC*(*−*) strains to observe the relative abundance of envelope proteins. Envelope proteins from both the strains were enriched by ultracentrifugation as described in Methods and quantitative proteomics was performed by iTRAQ. Two different experiments yielded a total of 760 and 335 proteins, respectively, including 225 proteins that were commonly identified by iTRAQ in the WCE and envelop fraction (Supplementary Data 2 and 3). A comparison of the differentially expressed proteins in the WCE and envelop further reveals that together with other proteins quite a few respiratory proteins such as putative succinate dehydrogenases Rv0247c and Rv0248c, ATP synthases AtpH and AtpG, and ubiquinol-cytochrome C reductase subunit QcrA are relatively less expressed (by <0.5-fold) in envelope than in WCE after YidC depletion (Fig. 5a and Supplementary Data 3). A comparative expression analysis indicates that with respect to control Mtb, levels of Rv0247c, Rv0248c, AtpH, AtpG and QcrA in envelope of *yidC*(*−*) are 0.32, 0.5, 0.37, 0.5 and 0.49-folds after normalization to their respective levels in the WCE. Remarkably, exogenous expression of Rv0248c-FLAG followed by anti-FLAG immunoblotting also indicated ~40% reduction in the expression of Rv0248c in the envelope of *yidC*(*−*) normalized to its levels in the cytoplasmic fraction (Supplementary Fig. 4b), thus validating the above results. While the expression of *atpH* and *atpG* transcripts are also modulated, the mRNA transcript levels of other three genes (*Rv0247c, Rv0248c* and *qcrA*) are not altered in *yidC*(*−*) (Fig. 5b), suggesting that silencing the YidC particularly affects expression of the Rv0247c, Rv0248c and QcrA proteins, and not their corresponding mRNAs. Overall the above results clearly demonstrate the involvement of YidC in translocation of respiratory proteins to cell envelope.

### YidC interacts with respiratory proteins

**Figure 6:**
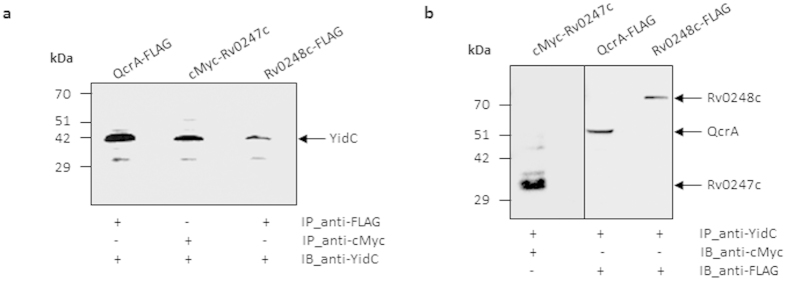
Immunoblot analyses to assess interaction of YidC with respiratory proteins. (**a**,**b**) *M. smegmatis* lysate expressing YidC of Mtb was incubated individually with QcrA-FLAG, cMyc-Rv0247c and Rv0248c-FLAG-expressing lysates for 16 hours followed by immunoprecipitation (IP) using anti-FLAG, anti-cMyc or anti-YidC antibodies, respectively. The immunoprecipitates of anti-FLAG or anti-cMyc antibodies were subjected to immunoblotting (IB) by anti-YidC (**a**), whereas those immunoprecipitated by anti-YidC were probed by anti-FLAG or anti-cMyc antibodies (**b**), respectively. Blots were developed by using clean-blot IP detection kit (Thermo Pierce) to avoid signals from denatured IgG subunits present in the immunoprecipitated samples. Data represent two experiments in (**a**,**b**).

Next, we examined if YidC is associated with the respiratory proteins Rv0247c, Rv0248c and QcrA in Mtb. All the three proteins were expressed in *M. smegmatis* either with N-terminal cMyc- (Rv0247c) or with C-terminal FLAG- (Rv0248c and QcrA) tags. Interaction of YidC with these proteins was subsequently analyzed by incubating WCEs of *M. smegmatis* expressing Mtb YidC protein with the cell lysates containing cMyc-Rv0247c, Rv0248c-FLAG and QcrA-FLAG, respectively, in the presence of either anti-YidC (for co-immunoprecipitating YidC), anti-cMyc (for co-immunoprecipitating cMyc-Rv0247c) or anti-FLAG (for co-immunoprecipitating Rv0248c-FLAG and QcrA-FLAG) IgGs. The protein-antibody complexes were immobilized on protein A-agarose resin, which were subsequently eluted by boiling in the SDS-PAGE loading dye after thorough washing. As shown in Fig. 6a, immunoblotting of anti-cMyc or anti-FLAG co-immunoprecipitated products with anti-YidC antibodies detects YidC in all the three samples, indicating the association of YidC with Rv0247c, Rv0248c and QcrA proteins. Interestingly, YidC was not detected in the cMyc-Rv0247c or QcrA-FLAG expressing cell lysates, when these were co-immunoprecipitated in the absence of Mtb YidC using respective antibodies (Supplementary Fig. 6, Lanes 1 and 2); these results thus clearly indicate that interaction of Rv0247c, Rv0248c and QcrA is specific to only Mtb orthologue of YidC. Moreover, presence of cMyc-Rv0247c, Rv0248c-FLAG and QcrA-FLAG in the anti-YidC co-immunoprecipitated products further confirms the specificity of interaction between YidC and the respective respiratory proteins (Fig. 6b).

### Expression of YidC is regulated by membrane potential

**Figure 7:**
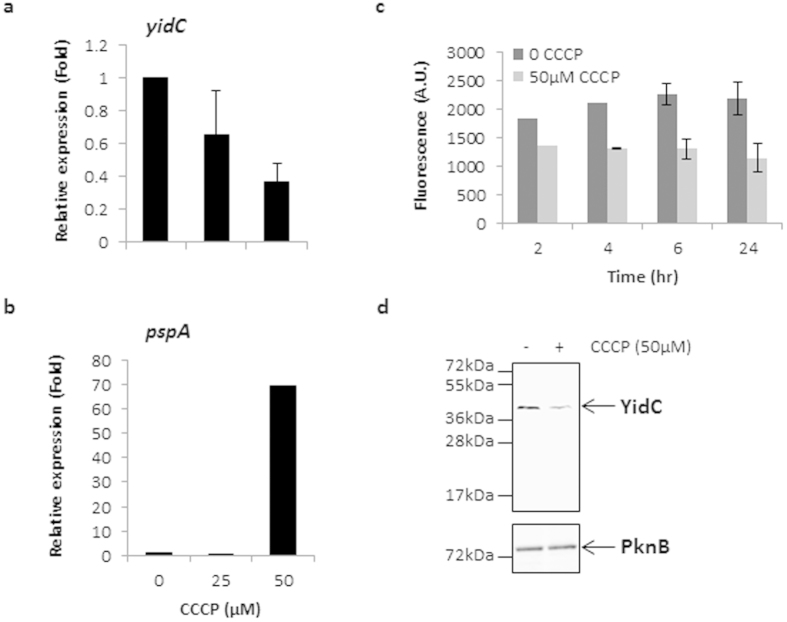
Effect of membrane potential on expression of YidC. (**a**,**b**) qRT-PCR studies were performed to assess the effect of ionophore CCCP on the expression of *yidC* (**a**) and a control gene *pspA*, which is induced by CCCP treatment^44^ (**b**). Briefly, Mtb cultures were incubated with 25 and 50 μM CCCP for 2 hours followed by RNA isolation and cDNA synthesis. Equal amounts of cDNAs were subsequently used for qRT-PCR by amplifying ~200 bp region of respective ORFs using specific forward and reverse primers (Supplementary Table 2). Data were obtained after normalization to *sigA* transcript levels, which remained constant in both the CCCP-treated and untreated cultures. Mean ± s.d. of three measurements is shown in (**a,b**). (**c**,**d**) Effect of 50 μM CCCP treatment on YidC expression was also observed either in Mtb expressing YidC-GFP at different time points by estimation of fluorescent intensities (**c**), or in wild-type bacteria after 6 hours, by anti-YidC immunoblotting of 20 μg proteins from envelope fraction (**d**). Mean ± s.d. of three measurements is shown in (**c**); data represent two experiments in (**d**). Overall, these results demonstrate that expression of YidC is regulated by membrane potential in Mtb.

Previously it is shown that disruption of membrane potential by ionophore carbonyl cyanide m-chlorophenylhydrazone (CCCP) perturbs the expression of respiratory genes in Mtb^42^. Moreover, CCCP treatment also affects the translocation of several membrane proteins in *Bacillus subtilis*^43^. Since YidC is an envelope protein which controls membrane potential and subsequent cellular ATP levels in Mtb, we analyzed whether expression of YidC itself is regulated by membrane potential. Wild-type Mtb cultures were treated with different concentrations of CCCP for two hours followed by mRNA expression analysis by qRT-PCR. It was observed that maintenance of a membrane potential is required for the expression of *yidC* in Mtb. Cells treated with CCCP exhibited a dose-dependent suppression of *yidC*, whereas the expression of *pspA*, which encodes a membrane stress protein, is enhanced by ~80-fold after 50 μM of CCCP treatment, as reported earlier^44^ (Fig. 7a,b). Further, we analyzed the effect of treatment with 50 μM CCCP for different time points on the expression of YidC-GFP fusion protein in Mtb. Expression was analyzed by measuring fluorescence at different time points which indicated a decline in the YidC-GFP expression by CCCP at all time points (Fig. 7c). A similar effect was also observed on the expression of native YidC in wild-type Mtb after 6 hrs of CCCP treatment (Fig. 7d). Taken together, these results indicate that expression of YidC is regulated by membrane potential in mycobacteria.

### YidC is required for intracellular survival of Mtb

**Figure 8:**
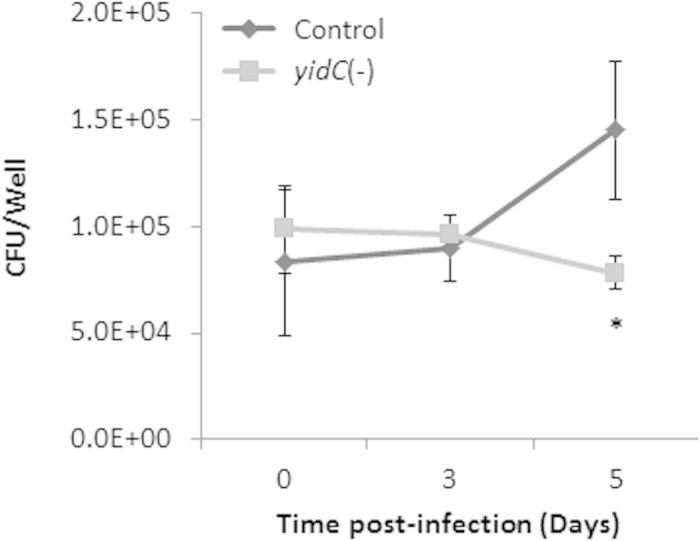
Role of *yidC* in intracellular survival of Mtb. Survival profile of the different Mtb strains in human monocyte-derived macrophages, THP-1. Macrophages were infected with empty plasmid-containing (control) or YidC-depleted (*yidC*(*−*)) Mtb at an MOI of 1:5. After lysis, intracellular mycobacteria were enumerated at different time points post-infection by cfu counting. Despite similar cfu counts of control and *yidC*(*−*) strains after 4 hours of infection, a substantial growth of control and not of *yidC*(*−*) strain over a period of 5 days indicates the requirement of YidC for intracellular survival of Mtb. The results are expressed as cfu/well in a 6-well plate. Mean ± s.d. of cfu counts from three wells is shown. Asterisk describes level of significance (*P* < 0.05).

Role of YidC in regulating the central metabolic pathways and respiration substantiates its requirement for growth of Mtb in the culture medium^37^. Recently, we showed that suppression of *yidC* for >4 days significantly arrests the *in vitro* growth of Mtb^37^. Importantly, we did not observe any change in cell morphology of Mtb *yidC*(*−*) strain. In order to further understand the requirement of YidC for intracellular survival of Mtb, THP-1 human monocyte-derived macrophages were infected with mid-log grown cultures of control and *yidC*(*−*) strains at an MOI of 1:5 for 4 hrs. Subsequently, intracellular survival of mycobacteria was monitored by CFU plating of macrophage lysates at different intervals. As may be seen in Fig. 8, *yidC*(*−*) exhibits poor growth in THP-1 macrophages in comparison to the control Mtb. While the control Mtb cells show a substantial increase in bacterial counts after 3 and 5 days of infection (8.30-, 9.00- and 14.55 × 10^4^ CFUs/well on days 0, 3 and 5, respectively), growth of *yidC*(*−*) within macrophages is essentially ceased (9.85-, 9.65 and 7.80 × 10^4^ CFUs/well on days 0, 3 and 5, respectively). These results thus clearly demonstrate that YidC is required for intracellular survival of Mtb in host macrophages.

## Discussion

In last two decades YidC–Oxa1–Alb3 family proteins have been extensively studied in different bacteria and eukaryotic organisms. Unfortunately, there is no information on role of YidC in mycobacterial pathogens. While YidC orthologues within Gram-positive and Gram-negative groups exhibit substantial similarity, it is astounding to note that Rv3921c-encoded YidC of Mtb exhibits >80% differences with its counterparts of either the Gram-positive (such as *B. halodurans* (Supplementary Fig. 1)) or Gram-negative bacteria. However, presence of YidC orthologues in different mycobacterial species including *M. leprae*, which contains minimal gene sets due to reductive evolution, suggests an important role of this protein in mycobacteria. Recently we observed that depletion of YidC in Mtb by clustered regularly interspaced short palindromic repeat-interference (termed as CRISPRi) approach led to a significant reduction in bacterial growth^37^, further proposing a crucial role of this protein in Mtb.

Despite exhibiting a great deal of differences with YidC2 of *B. halodurans*, Rv3921c-encoded putative YidC protein contains many such residues that are critical for the translocase activity of YidC2 such as R52, Q62, Q115, N211, N262 and Q269 (corresponding to R72, Q82, Q142, Q187, N248, and Q254, respectively, in YidC2) (Supplementary Fig. 1)^18^. Crystal structure of YidC2 has revealed that these residues, spread over the cytoplasmic halves of TM1-5, loosely interact with each other to form a hydrophilic groove of ~2000 Å^3^ in the lipid bilayer which is open to both the cytoplasmic side and the membrane interior, whereas the extracellular side is sealed by the hydrophobic core^18^. Notably, the R72 residue in YidC2 (corresponding to R52 of Mtb YidC) is the only charged residue that creates a strong positive electrostatic potential in the groove, and affect the membrane insertion of the substrate protein^18^. Additionally, YidC of Mtb also contains PXXG motif starting at P105 and ending at G108 residues in the TM3, which is characteristically present in channel proteins and plays a role in increasing their flexibility^45^. These initial observations together with localization of YidC to cell envelope of Mtb (Fig. 1) strongly suggest a translocase activity in this protein.

To understand the function of YidC in Mtb, we performed the transcriptomic and proteomic studies using *yidC*(*−*) strain of Mtb which lacks YidC. Whole genome transcriptional analysis by microarray indicated altered expression of ~10% of total Mtb genes in *yidC*(*−*), majority of which control EHR, respiration and central metabolic pathways (Fig. 2). The EHR genes include a total of 230 genes that respond to prolonged hypoxia and are critical for bacterial adaptation to multiple stresses such as transition between respiring and non-respiring states and changes in redox status during latency, an energy-conserving growth stage which requires less ATP^38^,^46^ (Fig. 2b). Perturbation of genes encoding ATP synthases and other respiratory proteins that are modulated by cellular ATP levels^40^ suggests a possible role of YidC in managing the ATP production in Mtb (Fig. 2c). Interestingly, the proteomic profile of Mtb lacking YidC significantly resembles to that exhibited by Mtb treated with ATP synthase inhibitor, bedaquilline^40^. Both, depletion of YidC and bedaquilline treatment affect intermediate metabolism and respiration in Mtb. Similar to the effect of bedaquilline, repression of YidC also results in accumulation of ATP synthase complex and components of stress response such as the heat shock protein (HspX) and bacterioferritin (BfrA), and strong downregulation of ribosomal proteins (Fig. 3). Taken together, the transcriptomic and proteomic profiles of *yidC*(*−*) clearly indicated the role of YidC in ATP synthesis. A significant decrease (~40%) in ATP concentration in Mtb depleted with YidC further substantiates our hypothesis that YidC is required to maintain the ATP levels in Mtb (Fig. 4a).

To gain a mechanistic insight into the function of YidC in Mtb, we first determined the effect of YidC depletion on the intracellular ratio of NADH, a coenzyme which is involved in redox reactions, to its oxidized form NAD^+^. As demonstrated in Fig. 4b, YidC depletion resulted in an elevated NADH/NAD^+^ ratio suggesting a redox imbalance in *yidC*(*−*). Concurrent increase in the expression of NDH-2 encoding gene, *ndh* and those belonging to electron acceptors (*cydC*, *cydD*, *cydB* and *cydA*) by feedback mechanism (Fig. 2c) implies a prospective defect in proton pump of Mtb lacking the expression of YidC. YidC has been implicated in the biogenesis of cytochrome c oxidase and formation of the F1F_o_-ATP synthase^20^. Relative expression analysis of proteins in the WCEs and envelope of both the control and *yidC*(*−*) strains of Mtb revealed that YidC might be involved in the translocation of succinate dehydrogenase subunits A_1 and B_1 (Rv0248c and Rv0247c, respectively) and ubiquinol-cytochrome C reductase subunit QcrA to cell envelope (Fig. 5). A direct interaction of YidC with these proteins indeed confirms our hypothesis and establishes for the first time a distinct role of YidC in biogenesis of respiratory chain complexes in Mtb (Fig. 6).

The flexibility of membrane-bound respiratory system in pathogens helps them optimizing energy production under variable environmental conditions including intracellular survival in the host^47^. In Mtb, it has remained largely unknown how the core proteins engaged in ATP synthesis are transported to membrane. In this report we describe for the first time the role of YidC in maintaining the cellular ATP levels in Mtb, possibly by regulating the membrane translocation of respiratory chain complexes that are engaged in energy generation. Proteins involved in respiration and electron transport have been extensively exploited as anti-TB drug targets in the recent years^48^,^49^. Since, YidC is highly conserved in Mtb complex bacteria (Supplementary Fig. 7) and is indispensable for intracellular bacterial growth (Fig. 8) our results propose YidC a novel anti-TB drug target.

## Methods

### Culturing of mycobacteria and subcellular fractionation of WCE

Bacterial strains and plasmids used in this study are summarized in Supplementary Table 1. Mycobacteria were cultured typically as described earlier^37^. Construction of *yidC*(*−*) strain is reported previously^37^. Knockdown strain of Mtb for conditional suppression of Rv3920c was generated by co-expressing the respective sgRNA (Supplementary Table 2) with dCas9, as described earlier^37^. Subcellular fractionation of WCEs was performed by following a protocol described by Dahl *et al.*^50^. Briefly, WCEs of Mtb were prepared by bead-beat lysis and 1 mg lysates were centrifuged at 100000 X *g* for 4 hours at 4 °C to pellet cell envelope. The resulting supernatant was collected and used as cytoplasmic fraction. The cell envelope fraction was washed twice with PBS and re-suspended in 100 μl of PBS containing 1% Triton-X-100 for overnight at 4 °C. Protein concentration in each fraction was estimated by BCA.

### Cloning of ORFs in pTetR

For expression of GFP, DNA sequence corresponding to ORF of *gfp* was cloned in pTetR plasmid at NdeI and HindIII sites. To achieve this, plasmid pBEN (kind gift from Dr. William Bishai, Johns Hopkins University, USA) containing 717 bp-long *gfp* sequence was subjected to mutagenesis to replace internal NdeI site 229CATATG234 with 229CACATG234 in the coding sequence of *gfp*, followed by its amplification by PCR using Pr1-Pr2 primer pair. The primer Pr1 was designed in a way such that it incorporates at 5′ end, the NdeI and PacI restriction sites followed by 27 bases encoding 9 glycine residues before the start codon of GFP, whereas Pr2 incorporates a HindIII site at the 3′ end. The PCR amplicon was subsequently cloned at NdeI and HindIII sites in pTetR resulting in a plasmid, pTetR-GFP. To express YidC in fusion with GFP at its C-terminus (YidC-GFP), a 1098 bp sequence corresponding to *yidC* ORF (excluding the stop codon) was PCR amplified from Mtb genomic DNA using Pr3 and Pr4 primers and cloned in pTetR-GFP at NdeI and PacI sites, resulting in a plasmid pTetR-YidC-GFP. Both pTetR-GFP and pTetR-YidC-GFP were used to transform *M. smegmatis* and expression of GFP and YidC-GFP was obtained after treatment of bacteria with 50 ng/ml ATc for 24 hours. In order to conditionally overexpress YidC in mycobacteria, the 1101 bp-long coding sequence of *yidC* was PCR amplified using primer pair Pr3-Pr5 followed by cloning in pTetR at NdeI and HindIII sites. Expression was analyzed in *M. smegmatis* containing pTetR-YidC after 24 hours of treatment with 50 ng/ml ATc.

To obtain the expression of cMyc-Rv0247c, Rv0248c-FLAG and QcrA-FLAG in *M. smegmatis* the corresponding ORFs were PCR amplified using primer pairs Pr6-Pr7, Pr8-Pr9 and Pr10-Pr11, respectively, such that *Rv0247c* and *qcrA* amplicons contain NdeI-XbaI sites, whereas the *Rv0248c* amplicon contains HindIII-XbaI sites. These ORFs were subsequently cloned at the respective sites in pTetR. For expression of proteins in fusion with FLAG tag at the C-terminus, complementary oligonucleotides bearing the coding and noncoding sequences of FLAG (Pr11-Pr12) were annealed and cloned in pTetR-Rv0248c or pTetR-QcrA at XbaI site. Similarly, for expression of cMyc-Rv0247c fusion protein the Pr13 and Pr14 oligonucleotides were annealed and cloned in pTetR-Rv0247c at NdeI site. *M. smegmatis* was transformed with the respective recombinant plasmids, pTetR-Rv0248c-FLAG, pTetR-QcrA-FLAG or pTetR-cMyc-Rv0247c, respectively and expression was induced by ATc treatment, as described above. The sequences of all the oligonucleotides used for constructing the above plasmids are listed in Supplementary Table 2.

### Microarray expression analysis

Microarray was performed typically as described earlier^37^. Total RNA was extracted by using Trizol (Invitrogen Life Technologies) from the control and *yidC*(*−*) strains of Mtb after 4 days of incubation with 50 ng/ml ATc and subjected to cDNA synthesis. For each hybridization of labeled probes on microarray slides containing 13768 oligonucleotides encompassing three sets of 3924 genes of Mtb (Microarrays Inc), the cDNA probes labelled with alexa fluor 555 or alexa fluor 647 (Invitrogen Life Technologies) were used in pairs. RNA samples were prepared in duplicate for hybridizing twice through reverse labeling of the respective cDNAs. Scanning of slides and data analyses were accomplished as described previously^37^.

### Proteome analysis by iTRAQ

- Tryptic digestion of proteins. For tryptic digestion, 100 μg proteins from control and *yidC*(*−*) strains were cleaned up by acetone precipitation. The pellets were suspended in 50 mM triethyl ammonium bicarbonate, pH 8.0 and denatured by adding 0.1% SDS. Subsequently, 5 mM (Tris-(2-carboxyethyl) phosphine was added in each sample and incubated at 60 °C for 1 hour to reduce the proteins followed by alkylation with 8.4 mM iodoacetamide for 30 minutes in dark. Each of the two samples was digested with 4 μg trypsin for overnight at 37  °C.
- Labelling of peptides. For iTRAQ labelling, peptides from 2 biological replicates of control and *yidC*(*−*) strains were incubated with 4-plex iTRAQ labelling reagents (AB SCIEX), according to manufacturer’s recommendations. After labeling, reaction was quenched by diluting the labeled samples in two volume of water. The resultant labeled peptide from control and *yidC*(*−*) strains were mixed together and dried under vacuum.
- Cation exchange fractionation of labeled peptides. The labeled peptide mixture was fractionated on Perkin Elmer Flexar HPLC system using Agilent Zorbax strong cation exchange (SCX) column (2.1 × 150 mm) having 5 μm particle size. Samples were dissolved in 80 μl of SCX loading buffer (8 mM ammonium formate (AF) and 30% acetonitrile (ACN)) and peptides were eluted using automatic fraction collector with linear gradient of AF (10 mM-500 mM) at the rate of 300 μl/minute at every minute for ~40 minutes. The eluted fractions (~40 fractions) were vacuum dried and reconstituted just before the second dimensional separation on C18 column on LC-MALDI for spotting.
- LC-MALDI separation and spotting of peptide. Separation and spotting of peptide samples were performed using Eksigent NanoLC 400 and Eksigent Ekspert LC spotter (AB SCIEX), respectively. The eluted samples were vacuum dried, reconstituted in 10 μl of 2% ACN and 0.1% formic acid in water and centrifuged at 10000 × *g* for 5 minutes. The clear supernatant was loaded onto a Cap-Trap C18 trap cartridge (Michrom BIosource Inc.) and desalted for 10 minutes at the rate of 10 μl/minute using 2% ACN and 0.05% trifluoroacetic acid (TFA) in water. Samples were separated on Chromolith Caprod RP-18e HR capillary column (150 × 0.1 mm; Merck Millipore) using linear gradient of buffer B (98% ACN and 0.05% TFA in water) in buffer A (2% ACN and 0.05% TFA in water). Peptides were eluted at the rate of 1.5 μl/minute, which were subsequently mixed with a matrix solution, α-cyano-4-hydroxycinnamic acid (5 mg/ml in 80% ACN and 0.1% TFA) and spotted at every 8 seconds.
- MALDI MS analysis of peptides. Mass spectrometry (MS) of offline spotted peptide samples was performed using the 5800 MALDI–TOF/TOF analyzer (AB SCIEX) and MS/MS analysis was done using 4000 Series Explorer software, version 4.0 (AB SCIEX). The Instrument was operated in positive ion mode and external calibration was performed using a calibration mixture 1 from mass calibration standard kit (AB SCIEX). The laser power was set between 3100 and 3500 for MS and between 3800 and 4300 for MS/MS acquisition. MS spectra were acquired between 800 to 4000 m/z. Glu-1 fibronectin peptide was used for MS/MS calibration of the spectra. After screening all LC-MALDI samples positioned in an MS–positive reflector mode using 1000 laser shot, the fragmentation of automatically selected precursors was performed at collision energy of 1 KV using air as collision gas with accumulation of 2500 shots from each spectrum. Up to 20 most intense ion signals per spot position having a signal to noise ration of >20 were selected as precursor for MS/MS acquisition in interpretation method.
- Peptide identification and quantification. Identification and quantification of peptides and proteins was performed by the ProteinPilot software version 4.0 (AB SCIEX) using Paragon algorithm as the search engine^51^. MS/MS spectra were searched against the Mtb-complex database of protein sequences downloaded from NCBI in FASTA format and incorporated in ProteinPilot database for search using a parameter of carbamidomethylation of cysteine residues. To estimate the false discovery rate (FDR), a decoy database search strategy was used. The FDR is defined as the percentage of decoy proteins identified against the total protein identification. The FDR was calculated by searching the spectra against the Mtb-complex database. The peptide selection criteria for relative quantification were performed as follows: only peptides unique for given protein were considered for relative quantification, excluding those common to other isoforms or proteins of the same family. Proteins were identified on the basis of having at least two peptides with an ion-score above 95% confidence.

### Estimation of cellular ATP level in Mtb

Bacterial cultures equivalent to OD_600_ of 1.0 were pelleted and re-suspended in 0.2 ml PBS followed by heat-lysis at 98 °C for 10 minutes. ATP was measured in cell lysates using Bac Titer-Glo^TM^ assay kit (Promega), as per the manufacturer’s recommendations.

### RT-PCR and quantification of mRNA transcripts

First strand cDNA synthesis was performed with 500 ng total RNA after DNase I-treatment using random hexamer primers and Superscript III RT (Invitrogen Life Technologies). PCR was performed with 50 ng cDNAs and SYBR Green PCR Master Mix (Applied Biosystems) using gene-specific primer pairs (Supplementary Table 2) and real-time quantification was carried out using the ABI 7500 Fast Real-Time PCR System (Applied Biosystems) as described by the manufacturer.

### Immunoblot analysis

Immunoblotting experiments with anti-YidC, anti-GroEL1 and anti-PknB were performed typically as described earlier^37^, whereas those with anti-FLAG (F3165, Sigma) or anti-cMyc (M4439, Sigma) were conducted according to the manufacturer’s specifications.

### Co-immunoprecipitation

For each co-immunoprecipitation experiment, 1 mg lysate of *M. smegmatis* expressing native or Mtb YidC was incubated with equal amount of WCEs containing the FLAG- or cMyc-tagged target proteins for 16 hours at 4 °C. The protein complexes were captured by further incubating the lysates for 16 hours at 4 °C with either 20 μg anti-YidC or 10 μg anti-FLAG/anti-cMyc IgGs. Immune complexes were collected by incubating samples with 10 μl Protein A agarose beads (Thermo Pierce) for 1 hour at 4  °C, followed by six washes with IP lysis buffer (Thermo Pierce) and boiling in SDS-PAGE loading dye. After SDS-PAGE and transfer to nitrocellulose membrane, the bound antigens were detected with specific antibodies by immunoblot analysis as described above.

### Infection of macrophages

THP-1 cells cultured in 6-well plates (Costar) at a concentration of 2 × 10^6^ cells per well in RPMI medium containing 10% heat-inactivated fetal bovine serum (FBS), were stimulated with 50 nM phorbol myristate acetate (Sigma) for overnight before infection. The media was removed and the cells were infected with gRNA-containing (control) or *yidC*(*−*) strains of Mtb at a multiplicity of infection (MOI) of 1:5 (macrophage:bacteria). After 4 hours, the macrophages were washed with PBS five times followed by the addition of RPMI-FBS medium containing 200 ng/ml ATc. At each time-point post-infection, cells from three wells were thoroughly washed followed by lysis in 0.1% Triton-X-100 (Sigma) for 5 minutes to release intracellular bacteria. The bacteria were serially diluted in PBS and plated on 7H11 agar plates for cfu estimation.

## Additional Information

**How to cite this article**: Thakur, P. *et al.* The preprotein translocase YidC controls respiratory metabolism in *Mycobacterium tuberculosis*. *Sci. Rep.* **6**, 24998; doi: 10.1038/srep24998 (2016).

## Supplementary Material

Supplementary Information

Supplementary Data 1

Supplementary Data 2

Supplementary Data 3
