# Supplementary material for: The preprotein translocase YidC controls respiratory metabolism in Mycobacterium tuberculosis
Source: Sci Rep. 2016 May 11;6:24998. doi: 10.1038/srep24998 (PMC4863248; doi:10.1038/srep24998)
Supplement: Supplementary Information [file srep24998-s1.pdf]

SUPPLEMENTARY INFORMATION

The preprotein translocase YidC controls respiratory metabolism in *Mycobacterium tuberculosis*

Preeti Thakur[1,2], Nagavara Prasad Gantasala[3], Eira Choudhary[1,4], Nirpendra Singh[3], Malik Zainul Abdin[2] and Nisheeth Agarwal*[1]

Keywords: Preprotein translocase; *Mycobacterium tuberculosis*; respiration, ATP synthesis.

***Corresponding Author**

Address for Correspondence:
Translational Health Science and Technology Institute,
NCR Biotech Science Cluster,
3rd Milestone, Faridabad–Gurgaon Expressway,
Faridabad- 121001 India

Affiliation:
1. Translational Health Science and Technology Institute,
NCR Biotech Science Cluster,
3rd Milestone, Faridabad–Gurgaon Expressway,
Faridabad- 121001 India

2. Faculty of Science,
Jamia Hamdard,
Hamdard Nagar,
New Delhi-110062, India

3. Regional Center for Biotechnology,
NCR Biotech Science Cluster,
3rd Milestone, Faridabad–Gurgaon Expressway,
Faridabad- 121001 India

4. Symbiosis School of Biomedical Sciences,
Symbiosis International University,
Lavale,
Pune- 412115 (Maharashtra) India

```
                  1                                                50
YidC2_BH      (1) MNYMKRRLLLFAGILLLVALAGCSTTDPITSESEGIWNHFFVYPMSWLIT
 YidC_MT      (1) -------------VSLLFDFFSLDFIYYPVSWIMWVWYRLFAFVLGPSN-

                  51                                              100
YidC2_BH     (51) TVANLLNGSYGLSIIIVTILIRLALLPLTLKQQKSMRAMQVIRPEMEAIQ
 YidC_MT     (37) ------FFAWALSVMFLVFTLRALLYKPFVRQIRTTRQMQELQPQIKALQ
                                       *         *

                  101                                             150
YidC2_BH    (101) KKYKEKGSKDPKVQQEMQKELLGLYQKHGVNPMAGCLPLFIQLPILMAFY
 YidC_MT     (81) KKYG-------KDRQRMALEMQKLQREHGFNPILGCLPMLAQIPVFLGLY
                                                           *

                  151                                             200
YidC2_BH    (151) FAIMRTEEIR----------------------------------------
 YidC_MT    (124) HVLRSFNRTTGGFGQPHLSVIENRLTGNYVFSPVDVGHFLDANLFGAPIG

                  201                                             250
YidC2_BH    (161) ------YHTFLWFDLGQPDYILP-----FVAGITTYFQFKMTMSHQQQMQ
 YidC_MT    (174) AYMTQRSGLDAFVDFSRPALIAVGVPVMILAGIATYFNSRASIARQSAEA
                                                       *

                  251                                             300
YidC2_BH    (200) KTNPSDSDNPMANMMQMQMKVMLYVMPVMIIIAGLSLPSALSLYWVIGNI
 YidC_MT    (224) AANP---------QTAMMNKLALYVFPLGVVVGGPFLPLAIILYWFSNNI
                                                                  *

                  301                                             350
YidC2_BH    (250) FMIIQTYFIVVKAPPLEVEQTK-----------QKSSKPNKA--------
 YidC_MT    (265) WTFGQQHYVFGMIEKEEEAKKQEAVRRRAANAPAPGAKPKRSPKTAPATN
                      *

                  351                                             400
YidC2_BH    (281) --------------------------------------------------
 YidC_MT    (315) AAAPTEAGDTDDGAESDASTERPADTSNPARRNSGPSARTPRPGVRPKKR
                  401
YidC2_BH    (281) --
 YidC_MT    (365) KR
```

Supplementary Figure 1. Comparative analysis of YidC homologous from *B. halodurans* and Mtb. Alignment of Mtb YidC (YidC_MT) with YidC2 of *B. halodurans* (YidC2_BH). Amino acid sequences of the two proteins were aligned using AlignX program of Vector NTI software. The number in parentheses before each sequence represents the position of amino acid residue in the alignment. The numbers at the top of the alignment are the positions of the multiple sequence alignment. Color codes for amino acid residues at a given position are as follows: 1) red: identical residues; 2) green: similar residues; 3) black: non-similar residues. Positions of the residues that constitute cytoplasmic groove in the YidC2_BH are marked by asterisks.

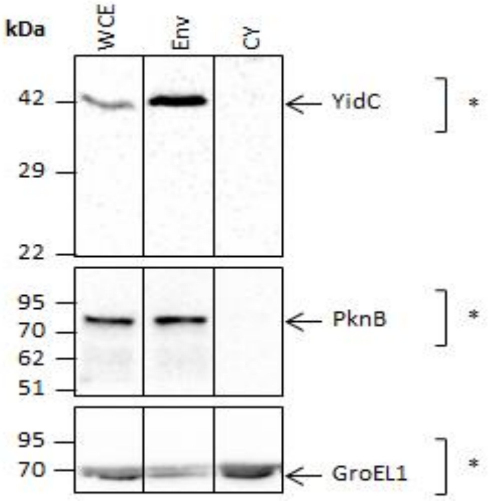

Supplementary Figure 2. Analysis of localization of YidC in Mtb by immunoblotting. Localization of YidC was analyzed in wild-type Mtb by subjecting the different subcellular fractions to anti-YidC immunoblotting. Equal amount of proteins from the whole cell extract (WCE), envelope (Env) and cytoplasm (CY) fractions were resolved on denatured polyacrylamide gel and blotted under the same experimental conditions. Antibodies against proteins that are destined to the Env (PknB) or both Env and CY (GroEL1) were simultaneously used to assess the purity of fractions. The immunoblot reveals that YidC is localized in the envelope and not in the cell cytosol. Asterisks represent blot regions shown in Figure 1d.

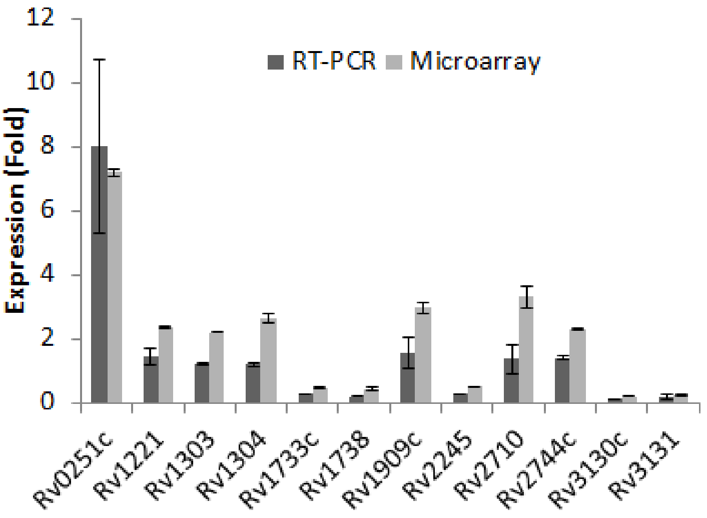

Supplementary Figure 3. Verification of differential gene expression analysis during microarray by qRT-PCR. Expression levels of randomly selected transcripts were analyzed by qRT-PCR in RNA preparations from control and *yidC(-)* strains used in microarray. Gene-specific primers amplifying ~200bp region of respective ORFs are listed in Table S1. Microarray expression values of the corresponding genes are plotted for a comparative analysis. Mean ± s.d. of two independent experiments is shown.

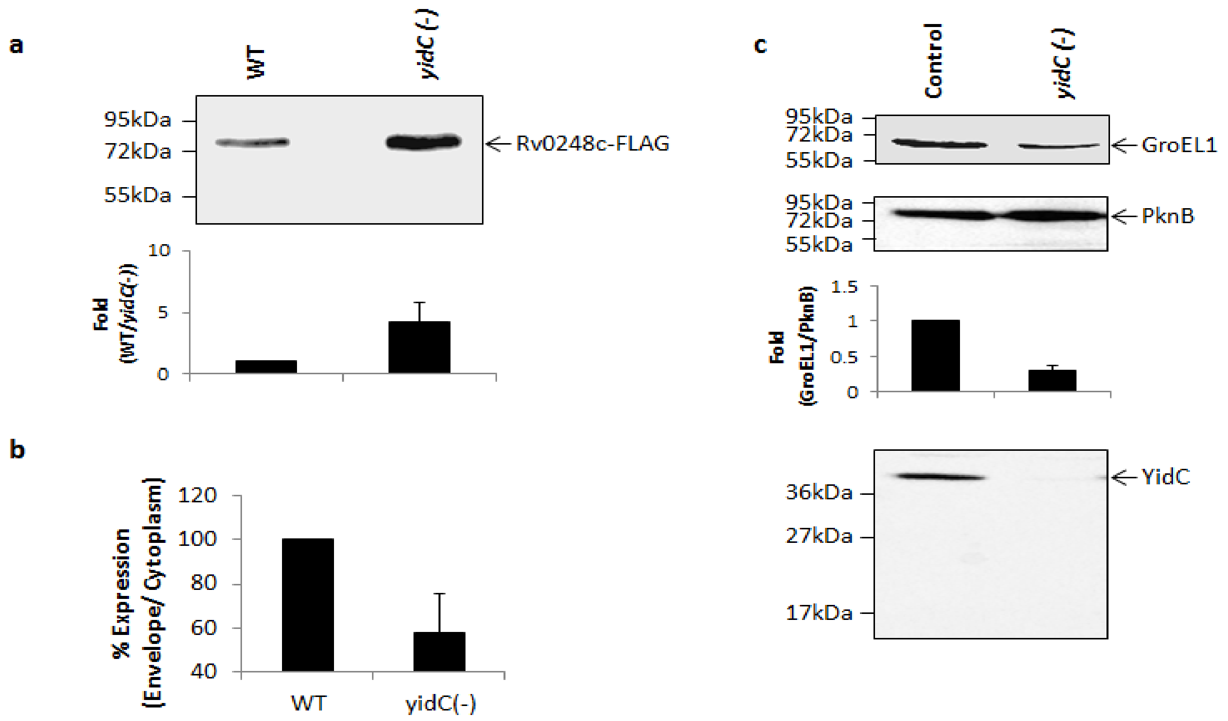

Supplementary Figure 4. Immunoblot analysis demonstrating the effect of YidC depletion on expression of proteins in Mtb. (**a**) Anti-FLAG immunoblotting with 20µg of WCE prepared from wild-type (WT) and *yidC(-)* strains harboring pTetR-Rv0248c-FLAG. As shown, suppression of YidC by 7 days of treatment with 50ng/ml ATc leads to ~4.2-fold more expression of Rv0248c-FLAG in the WCE. (**b**) Analysis of Rv0248c-FLAG expression levels in the envelope fraction of the WT and *yidC(-)* strains harboring pTetR-Rv0248c-FLAG, after normalization to its levels in the cytoplasmic fraction of the respective strains. Expression was determined by anti-FLAG immunoblotting with 10µg proteins, prepared after 7 days of treatment with 50ng/ml ATc. (**c**) Effect of YidC depletion on the expression of GroEL1. Shown are immunoblots with the WCEs of control and *yidC(-)* strains prepared after 7 days of treatment with 50ng/ml ATc probed with anti-GroEL1, anti-PknB and anti-YidC antibodies, respectively. A substantial decrease in the expression of YidC (>99%) and GroEL1 (~3-fold), but not of PknB in *yidC(-)* compared to control Mtb demonstrates a specific effect of YidC depletion on GroEL1 expression. Expression levels were quantitated by densitometric analysis, as presented by bar graph in (**a-c**). Data represent two independent experiments in (**a-c**).

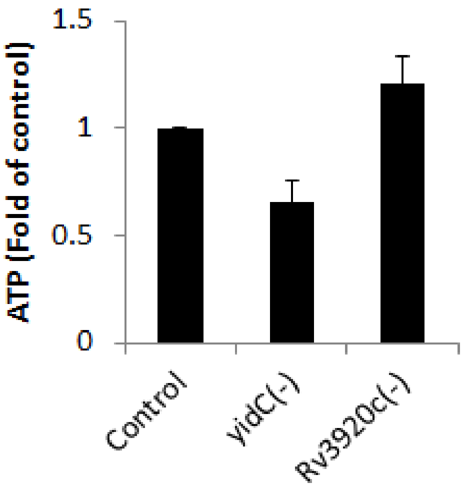

Supplementary Figure 5. Effects of depletion of Rv3920c on cellular ATP levels. ATP concentration was estimated after depletion of Rv3920c in Mtb, as described in Methods. Comparison of ATP levels in knockdown strain with control indicates no effect of suppression of Rv3920c on ATP production in Mtb. Mean ± s.d. of two experiments is shown.

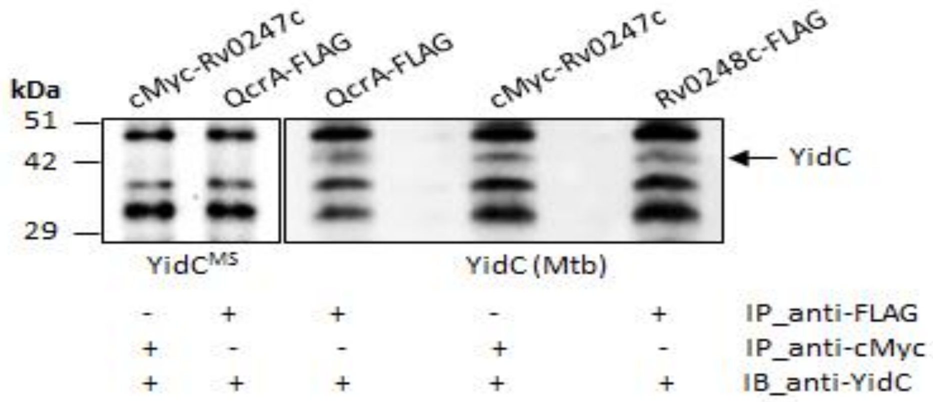

Supplementary Figure 6. Interaction of YidC^{MS} with QcrA and Rv0247c. *M. smegmatis* lysates expressing QcrA-FLAG or cMyc-Rv0247c were immunoprecipitated with anti-FLAG or anti-cMyc antibodies, respectively, followed by immunoblotting with anti-YidC antibodies. Absence of YidC in immunoprecipitated samples indicates no association of *M. smegmatis* YidC (YidC^{MS}) with any of the QcrA or Rv0247c proteins (lanes 1-2). In contrast, when immunoprecipitation of QcrA-FLAG, Rv0248c-FLAG or cMyc-Rv0247c-expressing lysates was performed in the presence of cell lysates expressing YidC of Mtb, YidC was detected in all the three samples (lanes 3-5), indicating specific interaction of Mtb YidC with these proteins. To confirm immunoprecipitation of IgGs, blots were developed with SuperSignal West Femto maximum sensitivity chemiluminescent substrate (Thermo Pierce), which also detected IgG heavy and light chains. Data represent two independent experiments.

```
                 1                                                                                                100
YidC_Maf    (1)  MSLLFDFFSLDFIYYPVSWIMWVWYRLFAFVLGPSNFFAWALSVMFLVFTLRALLYKPFVRQIRTTRQMQELQPQIKALQKKYGKDRQRMALEMQKLQRE
YidC_Mbo    (1)  MSLLFDFFSLDFIYYPVSWIMWVWYRLFAFVLGPSNFFAWALSVMFLVFTLRALLYKPFVRQIRTTRQMQELQPQIKALQKKYGKDRQRMALEMQKLQRE
YidC_Bcg    (1)  MSLLFDFFSLDFIYYPVSWIMWVWYRLFAFVLGPSNFFAWALSVMFLVFTLRALLYKPFVRQIRTTRQMQELQPQIKALQKKYGKDRQRMALEMQKLQRE
YidC_Mcan   (1)  MSLLFDFFSLDFIYYPVSWIMWVWYRLFAFVLGPSNFFAWALSVMFLVFTLRALLYKPFVRQIRTTRQMQELQPQIKALQKKYGKDRQRMALEMQKLQRE
YidC_Mcap   (1)  MSLLFDFFSLDFIYYPVSWIMWVWYRLFAFVLGPSNFFAWALSVMFLVFTLRALLYKPFVRQIRTTRQMQELQPQIKALQKKYGKDRQRMALEMQKLQRE
YidC_Mmi    (1)  MSLLFDFFSLDFIYYPVSWIMWVWYRLFAFVLGPSNFFAWALSVMFLVFTLRALLYKPFVRQIRTTRQMQELQPQIKALQKKYGKDRQRMALEMQKLQRE
YidC_Mtu    (1)  VSLLFDFFSLDFIYYPVSWIMWVWYRLFAFVLGPSNFFAWALSVMFLVFTLRALLYKPFVRQIRTTRQMQELQPQIKALQKKYGKDRQRMALEMQKLQRE

                 101                                                                                              200
YidC_Maf  (101)  HGFNPILGCLPMLAQIPVFLGLYHVLRSFNRTTGGFGQPHLSVIENRLTGNYVFSPVDVGHFLDANLFGAPIGAYMTQRSGLDAFVDFSRPALIAVGVPV
YidC_Mbo  (101)  HGFNPILGCLPMLAQIPVFLGLYHVLRSFNRTTGGFGQPHLSVIENRLTGNYVFSPVDVGHFLDANLFGAPIGAYMTQRSGLDAFVDFSRPALIAVGVPV
YidC_Bcg  (101)  HGFNPILGCLPMLAQIPVFLGLYHVLRSFNRTTGGFGQPHLSVIENRLTGNYVFSPVDVGHFLDANLFGAPIGAYMTQRSGLDVFVDFSRPALIAVGVPV
YidC_Mcan (101)  HGFNPILGCLPMLAQIPVFLGLYHVLRSFNRTTGGFGQPHLSVIENRLTGNYVFSPVDVGHFLDANLFGAPIGAYMTQRSGLDAFVDFSRPALIAVGVPV
YidC_Mcap (101)  HGFNPILGCLPMLAQIPVFLGLYHVLRSFNRTTGGFGQPHLSVIENRLTGNYVFSPVDVGHFLDANLFGAPIGAYMTQRSGLDAFVDFSRPALIAVGVPV
YidC_Mmi  (101)  HGFNPILGCLPMLAQIPVFLGLYHVLRSFNRTTGGFGQPHLSVIENRLTGNYVFSPVDVGHFLDANLFGAPIGAYMTQRSGLDAFVDFSRPALIAVGVPV
YidC_Mtu  (101)  HGFNPILGCLPMLAQIPVFLGLYHVLRSFNRTTGGFGQPHLSVIENRLTGNYVFSPVDVGHFLDANLFGAPIGAYMTQRSGLDAFVDFSRPALIAVGVPV

                 201                                                                                              300
YidC_Maf  (201)  MILAGIATYFNSRASIARQSAEAAANPQTAMMNKLALYVFPLGVVVGGPFLPLAIILYWFSNNIWTFGQQHYVFGMIEKEEEAKKQEAVRRRAANAPAPG
YidC_Mbo  (201)  MILAGIATYFNSRASIARQSAEAAANPQTAMMNKLALYVFPLGVVVGGPFLPLAIILYWFSNNIWTFGQQHYVFGMIEKEEEAKKQEAVRRRAANAPAPG
YidC_Bcg  (201)  MILAGIATYFNSRASIARQSAEAAANPQTAMMNKLALYVFPLGVVVGGPFLPLAIILYWFSNNIWTFGQQHYVFGMIEKEEEAKKQEAVRRRAANAPAPG
YidC_Mcan (201)  MILAGIATYFNSRASIARQSAEAAANPQTAMMNRLALYVFPLGVVVGGPFLPLAIILYWFSNNIWTFGQQHYVFGMIEKEEEAKKQEAVRRRAANAPAPG
YidC_Mcap (201)  MILAGIATYFNSRASIARQSAEAAANPQTAMMNKLALYVFPLGVVVGGPFLPLAIILYWFSNNIWTFGQQHYVFGMIEKEEEAKKQEAVRRRAANAPAPG
YidC_Mmi  (201)  MILAGIATYFNSRASIARQSAEAAANPQTAMMNKLALYVFPLGVVVGGPFLPLAIILYWFSNNIWTFGQQHYVFGMIEKEEEAKKQEAVRRRAANAPAPG
YidC_Mtu  (201)  MILAGIATYFNSRASIARQSAEAAANPQTAMMNKLALYVFPLGVVVGGPFLPLAIILYWFSNNIWTFGQQHYVFGMIEKEEEAKKQEAVRRRAANAPAPG

                 301                                                            366
YidC_Maf  (301)  AKPKRSPKTAPATNAAAPTEAGDTDDGAESDASTERPADTSNPARRNSGPSARTPRPGVRPKKRKR
YidC_Mbo  (301)  AKPKRSPKTAPATNAAAPTEAGDTDDGAESDASTERPADTSNPARRNSGPSARTPRPGVRPKKRKR
YidC_Bcg  (301)  AKPKRSPKTAPATNAAAPTEAGDTDDGAESDASTERPADTSNPARRNSGPSARTPRPGVRPKKRKR
YidC_Mcan (301)  AKPKRSPKTAPATNAAAPTEAGDTDDGAESDASTERPADTSNPARRNSGPSARTPRPGVRPKKRKR
YidC_Mcap (301)  AKPKRSPKTAPATNAAAPTEAGDTDDGAESDASTERPADTSNPARRNSGPSARTPRPGVRPRKRKR
YidC_Mmi  (301)  AKPKRSPKTAPATNAAAPTEAGDTDDGAESDASTERPADTSNPARRNSGPSARTPRPGVRPKKRKR
YidC_Mtu  (301)  AKPKRSPKTAPATNAAAPTEAGDTDDGAESDASTERPADTSNPARRNSGPSARTPRPGVRPKKRKR
```

Supplementary Figure 7. Alignment of YidC protein sequences from different bacteria of *M. tuberculosis* complex. Homologues of Mtb YidC (Mtu) were identified by Blastp homology searches in other Mtb complex bacteria such as *M. africanum* (Maf), *M. bovis* (Mbo), *M. bovis* BCG (Bcg), *M. canettii* (Mcan), *M. caprae* (Mcap) and *M. microti* (Mmi) that were aligned using AlignX program of Vector NTI software (Invitrogen) according to the manufacturer's recommendations. The number in parentheses before each sequence represents the position of amino acid residue of YidC protein sequence in the alignment. The numbers at the top of the alignment are the positions of the multiple sequence alignment. Color codes for amino acid residues at a given position are as follows: 1) red on yellow: identical residues; 2) black on green: block of similar residues; 3) blue on cyan: conserved residues; 4) green on white: residues weakly similar to consensus residue.

Supplementary Table 1. Bacterial strains and plasmids used in this study.

**A. Bacterial strains**			
**S. No.**	**Strain**	**Source**	
1.	*Mycobacterium smegmatis* mc^{2}155	Dr. William Jacobs, Albert Einstein College of Medicine, USA	
2.	*Mycobacterium tuberculosis* $H_{37}Rv$	Dr. William Bishai, Johns Hopkins University, USA	
3.	*Escherichia coli* DH5α	Dr. William Bishai, Johns Hopkins University, USA	
**B. Plasmids**			
**S. No.**	**Name**	**Description**	**Reference**
1.	pTetInt–*dcas9*	Mycobacteria- *E. coli* shuttle plasmid integrated into mycobacterial genome for the ATc inducible expression of dCas9.	37
2.	pTetR	Replicative Mycobacteria- *E. coli* shuttle plasmid for ATc inducible expression of different genes in mycobacteria.	37
3.	pGrna	Derivative of pTetR for ATc inducible expression of gene-specific guide RNA in mycobacteria.	37
4.	pGrna-*yidC*	Derivative of pGrna for ATc inducible expression of *yidC*-specific guide RNA.	37
5.	pGrna-*Rv3920c*	Derivative of pGrna for ATc inducible expression of *Rv3920c*-specific guide RNA.	This study
6.	pTetR-GFP	Derivative of pTetR for ATc inducible expression of GFP in mycobacteria.	This study
7.	pTetR-YidC-GFP	Derivative of pTetR for ATc inducible expression of YidC-GFP fusion protein in mycobacteria.	This study
8.	pTetR-YidC	Derivative of pTetR for ATc inducible expression of YidC in mycobacteria.	This study
9.	pTetR-Rv0248c-FLAG	Derivative of pTetR for ATc inducible expression of Rv0248c-FLAG fusion protein in mycobacteria.	This study
10.	pTetR-QcrA-FLAG	Derivative of pTetR for ATc inducible expression of QcrA-FLAG fusion protein in mycobacteria.	This study
11.	pTetR-cMyc-Rv0247c	Derivative of pTetR for ATc inducible expression of cMyc-Rv0247c fusion protein in mycobacteria.	This study

Supplementary Table 2. List of primers used in study.

S. No	Primer	Sequence (5'-3')	Reference
1	Pr1	CCGCATATGTTAATTAACGGTGGCGGTGGCGGTGGAGGTGGTGGGATGAGTAAAGGAGAAGAACTTTTC	Cloning of *gfp*
2	Pr2	GCCAAGCTTTTATTTGTATAGTTCATCCATGCCATG	
3	Pr3	GATCCATATGAGTCTTTTGTTTGATTTC	Cloning of *yidC* in pTetR-GFP
4	Pr4	GCGCTTAATTAAACGTTTGCGTTTTTTCGGTCGCACC	
5	Pr3	GATCCATATGAGTCTTTTGTTTGATTTC	Cloning of *yidC* in pTetR
6	Pr5	GCGCAAGCTTTCAACGTTTGCGTTTTTTCGGTC	
7	Pr6	GGCATATGACGTACAGCGCGAGTATGCG	Cloning of *Rv0247c* in pTetR
8	Pr7	GGTCTAGAGCGGCGAAACAGCTTGCTGC	
9	Pr8	AGAAGCTTGTTGAGGTCGAGCGGCACTC	Cloning of *Rv0248c* in pTetR
10	Pr9	GGTCTAGAGCCTCTCCGTCCTGGATGCTC	
11	Pr10	GGCATATGAGCCGCGCCGACGACGATG	Cloning of *qcrA* in pTetR
12	Pr11	GGTCTAGACTTAATTAATGTTGTTGTTCGCTCCCAGAATG	
13	Pr11	CTAGAGACTACAAGGACCACGACGGTGACTACAAGGACCACGACATCGACTACAAGGACGACGACGACAAGTGA	Cloning of FLAG gene expression cassette
14	Pr12	CTAGTCACTTGTCGTCGTCGTCCTTGTAGTCGATGTCGTGGTCCTTGTAGTCACCGTCGTGGTCCTTGTAGTCT	
15	Pr13	TAATGGAACAAAAACTTATTTCTGAAGAAGATCTGCA	Cloning of cMyc gene expression cassette
16	Pr14	TATGCAGATCTTCTTCAGAAATAAGTTTTTGTTCCAT	
17	Pr15	CTTCTGATGCACCGCCAAC	Cloning of Rv3920c-specific sgRNAs
18	Pr16	CGGTTGGCGGTGCATCAGAAGCATG	
19	Pr17	GTTTGATTTCTTCAGTCTCG	qRT-PCR analysis of *yidC*
20	Pr18	TTGATCTGTGGTTGCAGTTC	
21	Pr19	ATGGCCAATCCGTTCGTTAAAGC	qRT-PCR analysis of *pspA*
22	Pr20	GCATCTCCAATTGACGCTGG	
23	Pr21	CAATCTCGCATTGTGGTCGC	qRT-PCR analysis of *Rv0251c*
24	Pr22	TTGTCGACGTCAATGCCGGG	
25	Pr23	CGGGTTGGGAATACGGAATCG	qRT-PCR analysis of *Rv1221*
26	Pr24	CCTGCAATTGGTCAGACGGC	
27	Pr25	TGCCCTGCTGGTGCGGCGTTC	qRT-PCR analysis of *Rv1303*
28	Pr26	CGTCGTTGCCACCAGCAGCAC	
29	Pr27	CACCATTCAGATGCGCAATCAGG	qRT-PCR analysis of *Rv1304*
30	Pr28	ATTGATGTCCGCTGCTGCCG	
31	Pr29	GGTCTCGCTGCTGACTATCC	qRT-PCR analysis of *Rv1733c*
32	Pr30	TATTCCGTTCACGACCCATC	
33	Pr31	CATATCGATCGACGAACACG	qRT-PCR analysis of *Rv1738*
34	Pr32	GGGTCGACACCTTCAACATT	
35	Pr33	CCTCTATACCGGACTACGCC	qRT-PCR

36	Pr34	AGGGTTGGATCTTTCGCACC	analysis of *Rv1909c*
37	Pr35	ATCCACGCACTCGAAGACGAG	qRT-PCR analysis ofRv2245
38	Pr36	GAACCGGTCTGGATCGACCT	
39	Pr37	GGGTTGACAGCGATCTGGATGC	qRT-PCR analysis of *Rv2710*
40	Pr38	GGTCGCGTTTTCGGTTCTCG	
41	Pr39	ATGGCCAATCCGTTCGTTAAAGC	qRT-PCR analysis of *Rv2744c*
42	Pr40	GCATCTCCAATTGACGCTGG	
43	Pr41	GGATTCCGTGTCAATCCAAG	qRT-PCR analysis of *Rv3130c*
44	Pr42	GATGGTGACGTCGAATTTCC	
45	Pr43	GACCGACGAGGAATATCTGC	qRT-PCR analysis of *Rv3131*
46	Pr44	CGTCTGTCTCGGTGCCTAGT	
